# CHALLENGES IN ESTIMATING THE EFFECTIVENESS OF 2 DOSES OF COVID-19 VACCINE BEYOND 6 MONTHS IN ENGLAND

**DOI:** 10.1093/aje/kwad179

**Published:** 2023-09-01

**Authors:** Elsie M F Horne, William J Hulme, Ruth H Keogh, Tom M Palmer, Elizabeth J Williamson, Edward P K Parker, Venexia M Walker, Rochelle Knight, Yinghui Wei, Kurt Taylor, Louis Fisher, Jessica Morley, Amir Mehrkar, Iain Dillingham, Sebastian Bacon, Ben Goldacre, Jonathan A C Sterne, for the OpenSAFELY Collaborative

Understanding how the effectiveness of coronavirus disease 2019 (COVID-19) vaccine changes over time and in response to new severe acute respiratory syndrome coronavirus 2 (SARS-CoV-2) variants is crucial to scheduling subsequent doses. In a previous study, Horne et al. (1) quantified vaccine effectiveness (VE) over 6 consecutive 4-week periods from 2 weeks to 26 weeks after the second dose. Waning of hazard ratios (HRs) when comparing vaccinated persons with unvaccinated persons was approximately log-linear over time and was consistent across COVID-19–related outcomes and risk-based subgroups. To investigate waning beyond 26 weeks and in the era of the Omicron variant, we extended follow-up to the earliest of 50 weeks after the second dose or March 31, 2022.

## METHODS

The data source, study design, and statistical analysis are described in Web Appendix 1 and Web Table 1 (available at https://doi.org/10.1093/aje/kwad179). Ethical approval and data protection are detailed in Web Appendix 2. Eligible individuals were aged ≥18 years; registered at an English primary-care practice using TPP SystmOne (The Phoenix Partnership (Leeds) Ltd., Horsforth, United Kingdom); not in a residential care home (assisted living) or medically housebound; and had complete demographic data with no evidence of prior SARS-CoV-2 infection.

We estimated VE across 12 consecutive 4-week comparison periods in risk-based subgroups: persons aged ≥65 years, persons aged 18–64 years and clinically vulnerable (CV), persons aged 40–64 years, and persons aged 18–39 years. We estimated the VE of 2 doses of the BNT162b2 vaccine (Pfizer-BioNTech; Pfizer, Inc. (New York, New York) and BioNTech SE (Mainz, Germany)) and the ChAdOx1 vaccine (AstraZeneca; AstraZeneca AB, Cambridge, United Kingdom), versus no vaccine, in the age ≥65 years and age 18–64 years CV subgroups. VE could only be estimated for ChAdOx1 in the age 40–64 years subgroup and for BNT162b2 in the age 18–39 years subgroup.

Unvaccinated individuals were eligible for vaccination throughout follow-up. From the later of mid-September 2021 or 6 months after the second dose, individuals at highest risk of severe COVID-19 were offered a third dose (2, 3). Third-dose eligibility was progressively extended based on risk of severe COVID-19 until mid-December 2021, when concerns about the Omicron variant led to third doses being made available to all adults, with the required interval reduced to 3 months (4–6). In our VE models, unvaccinated individuals who received a first dose or vaccinated individuals who received a third dose were followed up for the remainder of that 4-week comparison period, then excluded. We fitted additional models to investigate factors associated with uptake of the third dose (Web Appendix 3).

## RESULTS

There were 1,990,562, 3,281,054, and 1,227,170 eligible individuals in the BNT262b2, ChAdOx1, and unvaccinated groups, respectively. Subgroup characteristics have been described previously (1). The earliest follow-up dates in the age ≥65, age 18–64 CV, age 40–64, and age 18–39 subgroups were March 15, April 21, May 18, and July 23, 2021, respectively. Individuals were followed for up to 50 weeks in the age ≥65 and 18–64 CV subgroups and up to 47 and 38 weeks in the age 40–64 and 18–39 subgroups, respectively. The latest follow-up date in all subgroups was March 31, 2022. Web Figure 1 shows the distribution of follow-up times per comparison period. Web Tables 2–21 show the number of events during each comparison period across subgroups and outcomes.

**Figure 1:**
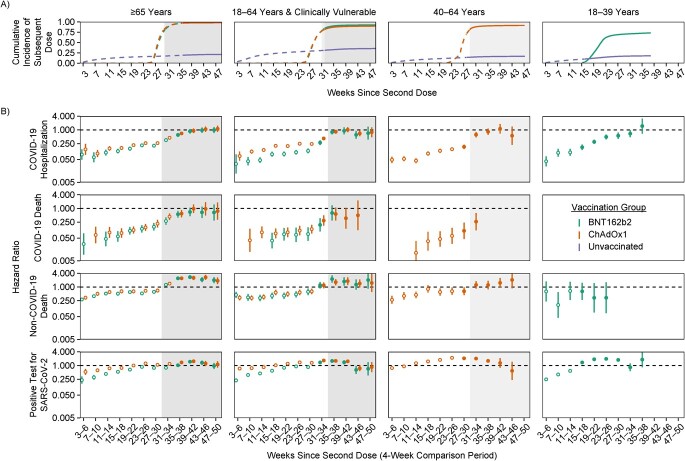
Cumulative incidence of a third dose of coronavirus disease 2019 (COVID-19) vaccine (A) and hazard ratios (HRs) for receipt of BNT162b2 (Pfizer-BioNTech) or ChAdOx1 (AstraZeneca) vaccine versus no vaccine (B) in England, 2020–2021. A) Cumulative incidence of receipt of a third dose in the vaccinated groups and receipt of a first dose in the unvaccinated groups throughout follow-up. Cumulative incidence curves are dashed before the Omicron variant became dominant and solid after it became dominant. B) HRs for BNT162b2 versus unvaccinated individuals and ChAdOx1 versus unvaccinated individuals. Circles are hollow before the Omicron variant became dominant and solid after it became dominant. HRs on the *y*-axes and estimated vaccine effectiveness are presented on the log scale. Each plot’s background is shaded where the cumulative incidence of receipt of a third dose was greater than 80%. Bars show 95% confidence intervals. SARS-CoV-2, severe acute respiratory syndrome coronavirus 2.

The cumulative incidence of receiving a third dose of vaccine increased rapidly during the 8 weeks following eligibility (Figure 1A). In the age ≥65 subgroup, incidence increased from 1% 23 weeks after the second dose to 93% or more by 31 weeks. Trends were similar in the age 18–64 CV and age 40–64 subgroups, reaching 90% or more. In the age 18–39 subgroup, incidence increased from 1% after 15 weeks to 62% after 23 weeks and 73% after 38 weeks. Uptake of a third dose was over 5 times lower in persons with (versus without) a recent positive SARS-CoV-2 test, and also lower in those who were in a hospital after unplanned admission, particularly if the admission included a COVID-19 code, and those who initiated end-of-life care (except the age 18–39 subgroup, in whom such events were rare; see Web Figures 2–5).

Because of high uptake of the third dose, the estimated effectiveness of 2 doses during later comparison periods was based on highly selected individuals who had received 2 but not 3 doses. Estimated HRs for non–COVID-19 death in the age ≥65, 18–64 CV, and 40–64 years subgroups changed markedly over the comparison periods during which most third doses were administered (Figure 1B). In the age ≥65 subgroup, estimated HRs comparing non–COVID-19 deaths among persons with 2 BNT162b2 doses versus no vaccine doses increased from 0.61 (95% confidence interval (CI): 0.51, 0.73) to 2.40 (95% CI: 2.02, 2.85) during weeks 27–30 and 35–38, respectively. Trends were similar for ChAdOx1 and the age 18–64 CV and 40–64 subgroups. Because estimated HRs for non–COVID-19 death strongly suggested selection bias arising from deferred vaccination in people with a recent SARS-CoV-2 infection or in poor health, we did not attempt to interpret estimated HRs beyond 26 weeks for COVID-19–related outcomes in the age ≥65, 18–64 CV, and 40–64 subgroups.

In the age 18–39 years subgroup, estimated HRs for non-COVID death (BNT162b2 only), although imprecisely estimated, did not change markedly during the rollout of third vaccine doses (Figure 1B). The cumulative incidence of a third dose was lower in this subgroup than in other subgroups, and postponement of vaccination because of ill health was rare. Waning of HRs for COVID-19 hospitalization was approximately log-linear over time, from 0.04 (95% CI: 0.03, 0.07) during weeks 3–6 to 1.48 (95% CI: 0.69, 3.17) by weeks 35–38. Waning of HRs for a positive SARS-CoV-2 test was approximately log-linear up to weeks 23–26 after the second dose. Estimated HRs were 0.25 (95% CI: 0.24, 0.26) during weeks 3–6, with HRs being greater than 1 by weeks 5–18. By weeks 23–26, the HR for a positive SARS-CoV-2 test (HR = 1.97, 95% CI: 1.91, 2.02) was close to the HR for any SARS-CoV-2 test (HR = 2.16, 95% CI: 2.12, 2.19). HRs for any SARS-CoV-2 test remained close to 2 throughout follow-up (Web Figure 6). Waning of HRs against a positive SARS-CoV-2 test and COVID-19 hospitalization in this subgroup did not appear to be affected by the emergence of the Omicron variant.

## DISCUSSION

Cumulative incidence of a third dose of COVID-19 vaccine in the age ≥65, 18–64 CV, and 40–64 years subgroups reached 90% or more. In these subgroups, vaccinated individuals who did not receive a third dose were at higher risk of non–COVID-19 death than unvaccinated individuals, due to postponement of vaccination because of SARS-CoV-2 infection or acute illness requiring an unplanned hospital admission. In these subgroups, estimates of the effectiveness of a second dose against COVID-19–related outcomes are unlikely to be meaningful beyond 6 months, because they are based on highly selected individuals. In these subgroups, it is difficult to disentangle the effect of the Omicron variant from depletion of the 2-dose group due to receipt of a third dose.

In the age 18–39 years subgroup, the maximum cumulative incidence of a third dose was 73%, and there was no evidence that individuals who remained in the 2–vaccine-dose group were at greater risk of non–COVID-19 death than unvaccinated individuals. Waning of HRs against COVID-19 hospitalization in this subgroup was approximately log-linear, and VE was negligible by weeks 35–38 after the second dose. Waning of HRs against a positive SARS-CoV-2 test was approximately log-linear until weeks 23–26, and VE was negligible by weeks 15–18. This finding should be interpreted with caution, as it may have been due to higher uptake and reporting of SARS-CoV-2 tests in vaccinated persons than in unvaccinated persons. Waning HRs in the age 18–39 group did not appear to be affected by emergence of the Omicron variant.

In an Australian survey, Glasziou et al. (7) found that unvaccinated individuals reported lower intentions to test for SARS-CoV-2 when symptomatic and lower intentions to report a positive SARS-CoV-2 test than vaccinated individuals. While estimated HRs reported here were adjusted for characteristics including previously reported SARS-CoV-2 tests (Web Table 1), unmeasured confounding by testing behavior probably remained given that HRs for any SARS-CoV-2 test were approximately 2 throughout follow-up. Waning of HRs for a positive SARS-CoV-2 test in the age 18–39 subgroup was approximately log-linear until weeks 23–26, and then plateaued and was close to the HRs for any SARS-CoV-2 test for the remaining comparison periods (except weeks 31–34). A tentative interpretation is that estimated VE against a positive SARS-CoV-2 test does not become negligible until weeks 23–26 (the inflection point in log-linear waning), while HRs greater than or equal to 1 were a result of uncontrolled confounding relating to differences in testing behavior between vaccinated and unvaccinated individuals. Follow-up from week 23 onward in this subgroup (Web Figure 1) coincided with changes in testing policy in early January 2022 (8) and the announcement in February that freely available mass testing would stop on April 1, 2022 (9). The end of follow-up for this study was March 31, 2022, but changes in testing behaviors are likely to have preceded this.

Third doses of COVID-19 vaccine should be deferred until 4 weeks after the start of a SARS-CoV-2 infection (10), consistent with our finding that uptake of a third dose was 5 times lower in persons with a recent positive SARS-CoV-2 test than in those without one (Web Appendix 3). Consequently, a high proportion of individuals remaining in 2–vaccine-dose groups after widespread uptake of the third dose may have had current or recent SARS-CoV-2 infection. Individuals who reported a positive SARS-CoV-2 test were removed from subsequent comparison periods where the outcome was SARS-CoV-2-test–related. However, they remained in subsequent comparison periods for all other outcomes. Thus, higher prevalence of a recent or current positive SARS-CoV-2 test in 2–vaccine-dose groups due to delayed vaccination could have resulted in higher rates of COVID-19 hospitalization or death and underestimates of VE against these outcomes following widespread uptake of third doses.

Researchers in previous studies have reported estimates of effectiveness of a second dose beyond 6 months (11–13) and reduced VE against the Omicron variant (11). However, the impacts of third-dose uptake on estimated second-dose VE, and of changes in testing policy and behaviors, are rarely discussed. This study demonstrated the importance of these factors in interpreting estimated VE. Studies increasingly focus on the incremental effectiveness of additional doses, rather than using unvaccinated individuals as the comparator. Investigators in such studies should carefully consider reasons why eligible individuals may not have received additional doses, particularly when the cumulative incidence of additional doses is high. We explored this by fitting models to investigate the baseline and time-updating characteristics associated with uptake of a third dose. However, HRs from these models may be biased by time-dependent confounding, so the results should not be interpreted as estimates of causal effects. Reporting of non–COVID-19 outcomes may also provide important insights into potential biases affecting interpretation of estimated VE.

It is challenging to estimate the long-term effectiveness of 2 COVID-19 vaccine doses in populations in which uptake of a third dose was high. These challenges also affect investigations of VE against the Omicron variant, whose emergence coincided with rapid uptake of third doses and of incremental effectiveness of a third dose against the second dose. Uptake of the third dose was sufficiently high that we do not believe that for the data analyzed here, much could be done to address the biases we have identified beyond constraining the time frames over which VE is estimated. However, in situations where uptake was more gradual, weighting observations by the inverse probability of censoring due to vaccination is a useful way to address informative censoring.

## Supplementary Material

Web_Material_kwad179
