# Supplementary material for: CHALLENGES IN ESTIMATING THE EFFECTIVENESS OF 2 DOSES OF COVID-19 VACCINE BEYOND 6 MONTHS IN ENGLAND
Source: Am J Epidemiol. 2023 Sep 1;193(1):227–31. doi: 10.1093/aje/kwad179 (PMC10773473; doi:10.1093/aje/kwad179)
Supplement: Web_Material_kwad179 [file web_material_kwad179.pdf]

WEB MATERIAL

Challenges in Estimating the Effectiveness of 2 Doses of COVID-19 Vaccine Beyond 6 Months in England

Elsie M. F. Horne, William J. Hulme, Ruth H. Keogh, Tom M. Palmer, Elizabeth J. Williamson, Edward P. K. Parker, Venexia M. Walker, Rochelle Knight, Yinghui Wie, Kurt Taylor, Louis Fisher, Jessica Morley, Amir Mehrkar, Iain Dillingham, Sebastian Bacon, Ben Goldacre, and Jonathan A. C. Sterne for the OpenSAFELY Collaborative

CONTENTS

Web Appendix 1 2

Web Appendix 2 4

Web Appendix 3 4

Web Figures 1–6 6

Web Tables 1–21 12

References 21

Web Appendix 1

This appendix describes the methods for estimating vaccine effectiveness. The following subsections (*Data source*, *Study design,* and *Statistical analysis*) are abridged versions of the published text describing our previous study, with added information on classification of dominant variant over time. See Horne *et al.* (2022) for full details.[1]

Data source

OpenSAFELY-TPP includes detailed pseudonymised primary care data linked (via National Health Service (NHS) number) with accident and emergency attendance, inpatient hospital spell records (NHS Digital's Hospital Episode Statistics dataset), national SARS-CoV-2 testing records (Second Generation Surveillance System; SGSS), and national death registry records. Vaccination status (National Immunisation Management System; NIMS) is available in the primary care record. Healthcare worker status (recorded for vaccine recipients at the time of vaccination) is provided by NHS Digital's covid-19 data store.

Study design

This study was approved by NHS England. People were eligible for inclusion if they were aged ≥18 years on 1 July 2021 and had been registered with a primary care doctor for at least one year before eligibility for their first vaccine dose (the "eligibility date"). People were excluded if they were aged >120 years on the date at which they became eligible for vaccination; their sex, geographical region, ethnicity, or English Index of Multiple Deprivation were unknown; or they were resident in a care home or medically housebound at six weeks after their eligibility date.

We defined three groups who received two doses of BNT162b2, received two doses of ChAdOx1, or were unvaccinated. Eligibility for the vaccinated groups was restricted to people who received their second vaccine dose during a four week "second vaccination period" within analysis strata defined by UK Joint Committee on Vaccination and Immunisation (JCVI) priority groups, eligibility date (for priority groups within which eligibility was based on age), and English NHS region (defined using individuals' primary care practice address).

We defined the second vaccination period as the four week period during which the greatest number of people in the stratum received their second dose. We excluded people from the vaccine groups if they received their first dose before their eligibility date, had an interval between first and second dose of less than six or more than 14 weeks, or were flagged as a healthcare worker on their vaccination record. We assigned people to the unvaccinated group if they had received no covid-19 vaccine at the start of the second vaccination period for their analysis stratum. We excluded people from any group if they had evidence of previous SARS-CoV-2 infection by the start of their second vaccination period (either a positive SARS-CoV-2 test in SGSS or probable covid-19 coded in primary care records), had ever been recorded as being resident in a care home, or had evidence of having started an end-of-life care pathway.

The analysis timescale was calendar time, which ensured that vaccinated and unvaccinated people were compared on the calendar day on which each outcome event occurred. We split follow-up time for fully vaccinated people into six consecutive four week "comparison periods," starting two weeks after receipt of the second dose. Because each second vaccination period was four weeks long and each vaccinated person was followed up for four weeks per comparison period, vaccinated people were followed during eight calendar weeks in each comparison period. Vaccinated people entered and finished follow-up on the calendar dates corresponding to the start and end of their comparison period.

We followed up unvaccinated people for the full eight calendar weeks that spanned the comparison periods for vaccinated people. To avoid overlap in follow-up of unvaccinated people between comparison periods, follow-up time for unvaccinated people was assigned at random to start either two or six weeks after the start of the second vaccination period and was split into the three consecutive eight week calendar periods during which vaccinated people were followed in each comparison period. Unvaccinated people assigned to start at two weeks were followed during odd numbered comparison periods 1, 3, 5, etc., and those assigned to start at six weeks were followed during even numbered comparison periods 2, 4, 6 etc.

We categorised follow-up time according to the dominant variant at that time, "dominant" defined as the percentage of cases with/without S-gene target failure exceeding 50%. Follow-up time before 31 May 2021 was categorised as "alpha", follow-up time from 1 June 2021 to 14 December 2021 was categorised as "delta", and follow-up time from 15 December 2021 onwards was categorised as "omicron". [2] Within subgroups, we categorised the comparison periods as alpha, delta or omicron based on the calendar period over which the majority of the follow-up time occurred (Web Figure 1).

Statistical analysis

For each comparison period, we estimated hazard ratios comparing BNT162b2 or ChAdOx1 recipients versus unvaccinated people. We did not estimate hazard ratios for comparison periods with fewer than three events in either group. For each person, follow-up ended at the earliest of the outcome of interest, deregistration from the primary care practice, death, or 31 March 2021. Fully vaccinated people who received a booster dose, and unvaccinated people who received a first dose were excluded from subsequent comparison periods, but follow-up within comparison periods was not censored after these events.

To estimate hazard ratios, we fitted Cox regression models with baseline hazards stratified by JCVI group, eligibility date, and region, and with the covariates described previously.[1] To avoid problems with model convergence, we excluded binary covariates from the model if any cell of the table defined by cross tabulating the covariate with vaccine group and comparison period contained fewer than three events. For categorical covariates with more than two levels, levels were merged until either all levels had more than three events or only one level existed, in which case the variable was excluded. We carried out this process independently for each outcome. We modelled age within strata as linear, with quadratic terms additionally included for strata with age range >5 years.

We did all analyses independently in four vaccine priority subgroups: aged ≥65 years and in JCVI groups 2-5, aged 18-64 years and clinically vulnerable (JCVI groups 4 or 6), aged 40-64 years (JCVI groups 7-10; restricted to those who received ChAdOx1), and aged 18-39 years (JCVI groups 11-12; restricted to those who received BNT162b2). The ≥65 subgroup included participants who were clinically vulnerable, whereas the 40-64 and 18-39 subgroups did not.

Web Appendix 2

This study was approved by the Health Research Authority (REC reference 22/PR/0095) and by the University of Bristol's Faculty of Health Sciences Ethics Committee (reference 117269).

NHS England is the data controller for OpenSAFELY-TPP; TPP is the data processor; all study authors using OpenSAFELY have the approval of NHS England. This implementation of OpenSAFELY is hosted within the TPP environment which is accredited to the ISO 27001 information security standard and is NHS IG Toolkit compliant;[3]

Patient data has been pseudonymised for analysis and linkage using industry standard cryptographic hashing techniques; all pseudonymised datasets transmitted for linkage onto OpenSAFELY are encrypted; access to the platform is via a virtual private network (VPN) connection, restricted to a small group of researchers; the researchers hold contracts with NHS England and only access the platform to initiate database queries and statistical models; all database activity is logged; only aggregate statistical outputs leave the platform environment following best practice for anonymisation of results such as statistical disclosure control for low cell counts.[4]

The OpenSAFELY research platform adheres to the obligations of the UK General Data Protection Regulation (GDPR) and the Data Protection Act 2018. In March 2020, the Secretary of State for Health and Social Care used powers under the UK Health Service (Control of Patient Information) Regulations 2002 (COPI) to require organisations to process confidential patient information for the purposes of protecting public health, providing healthcare services to the public and monitoring and managing the COVID-19 outbreak and incidents of exposure; this sets aside the requirement for patient consent.[5] This was extended in July 2022 for the NHS England OpenSAFELY COVID-19 research platform.[6] In some cases of data sharing, the common law duty of confidence is met using, for example, patient consent or support from the Health Research Authority Confidentiality Advisory Group.[7]

Taken together, these provide the legal bases to link patient datasets on the OpenSAFELY platform. GP practices, from which the primary care data are obtained, are required to share relevant health information to support the public health response to the pandemic, and have been informed of the OpenSAFELY analytics platform.

Web Appendix 3

This appendix describes the methods for investigating factors associated with uptake of third dose.

Study population

All individuals eligible for the vaccinated group in the vaccine effectiveness analysis.[1]

Statistical analysis

We fitted Cox models stratified by JCVI group and eligibility date for first vaccination dose. The time scale was time since second dose, and the outcome event was third dose (any brand). Follow-up time was split according to the time-updated covariates. We adjusted for the following covariates:

- Continuous covariates defined at baseline:
    - Age (second degree polynomial), date of second dose (natural cubic spline with three knots)
- Categorical covariates defined at baseline:
    - Sex (male, female), Index of Multiple Deprivation Quintile, Ethnicity (White, Black, South Asian, Mixed, Other), Body Mass Index (Not obese, 30.0-34.9, 35-39.9, 40+), brand of second dose (BNT162b2, ChAdOx1), number of SARS CoV-2 tests taken prior to first vaccination (0, 1, 2, 3+)
- Binary covariates defined at baseline:
    - Learning disability, severe mental illness, chronic respiratory disease, chronic heart disease, chronic liver disease, chronic kidney disease, chronic neurological disease, diabetes, immunosuppression, cancer (in the three years before baseline), flu vaccine (in the five years before baseline)
- Time-updated binary covariates:
    - End of life care initiated, positive SARS-CoV-2 test in the past 30 days, in hospital on a planned admission, in hospital on an unplanned admission, in hospital on an unplanned admission with a COVID-19 code

Web Figures 1-6

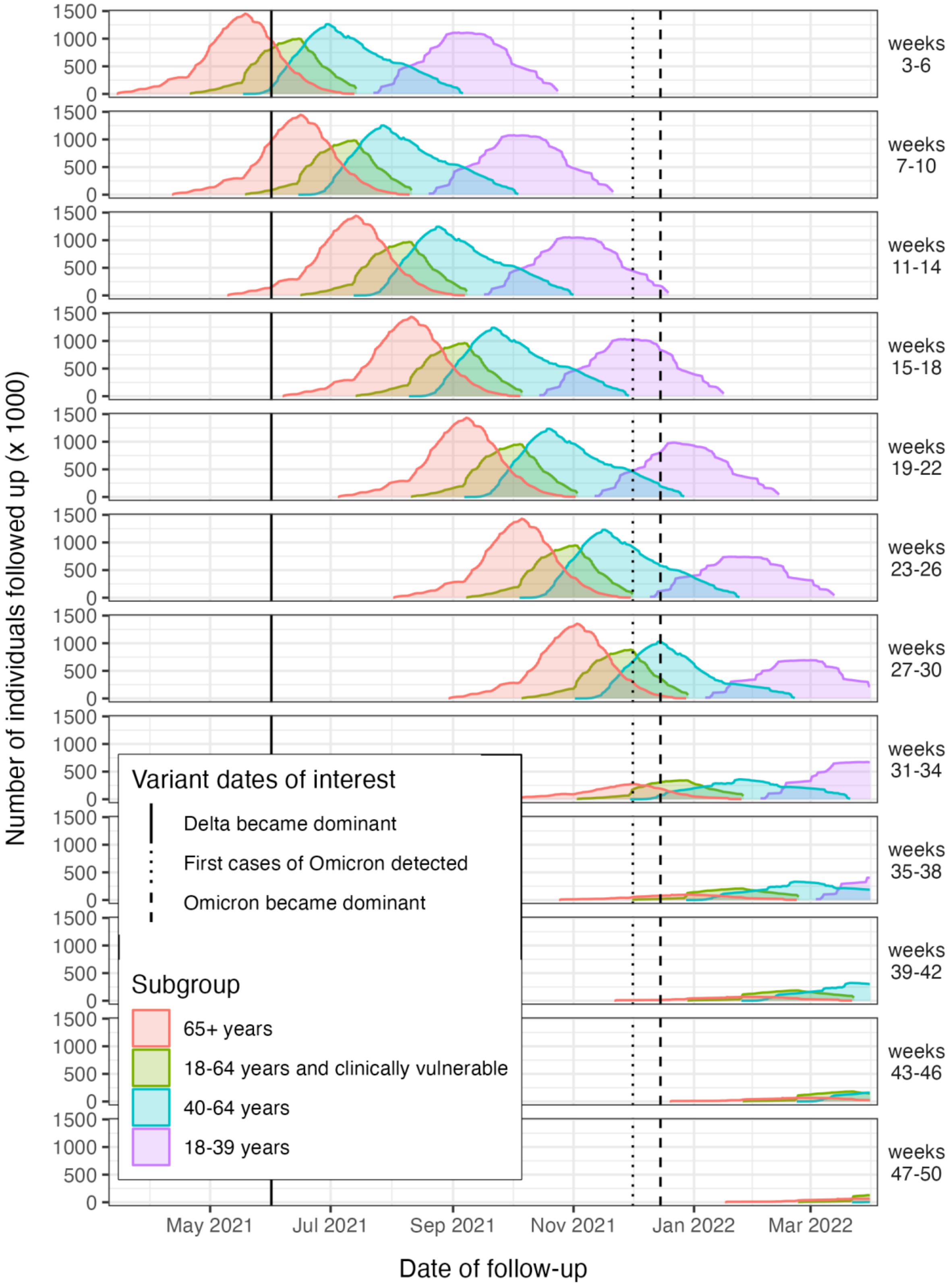

Web Figure 1. Distribution of follow-up time across comparison periods (defined by weeks since second dose)

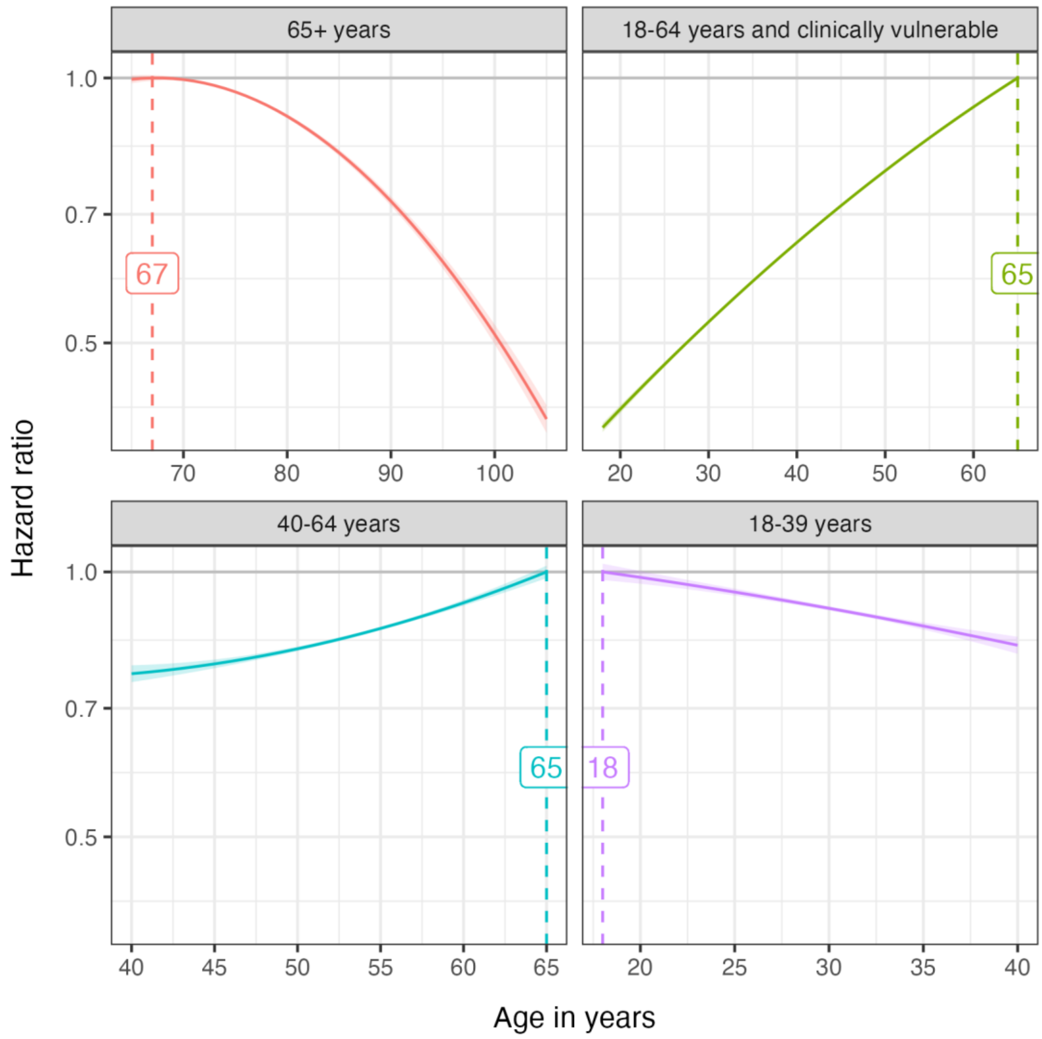

Web Figure 2. Hazard ratios for age, relative to the age with the highest predicted rate of third dose (shown by the dashed vertical line). Note that the subgroups were defined at the time of eligibility for first dose, so individuals may be up to one year older than the age range stated in the subgroup label.

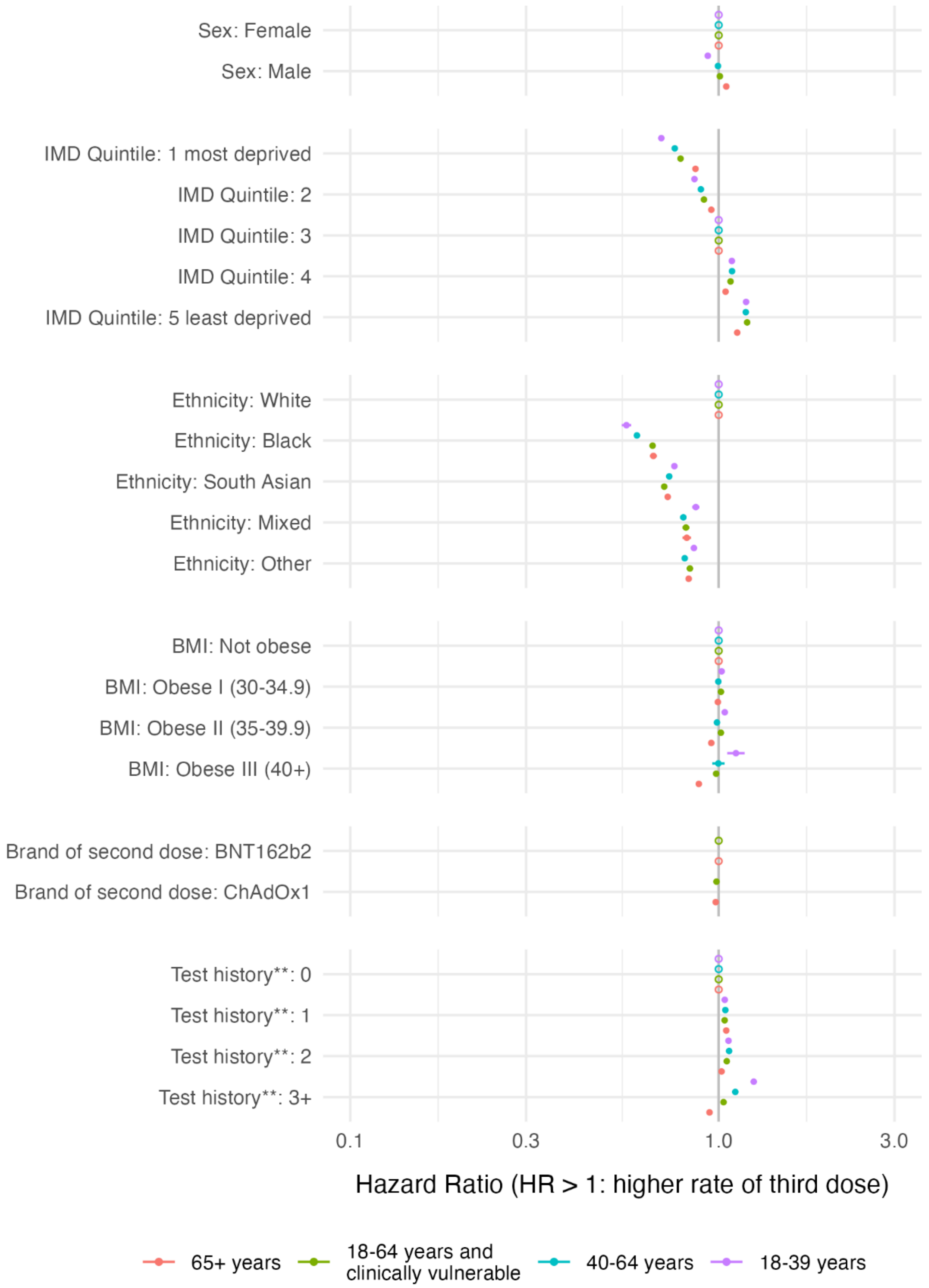

Web Figure 3. Hazard ratios for uptake of third dose. All variables defined at baseline. Hollow points correspond to reference categories.

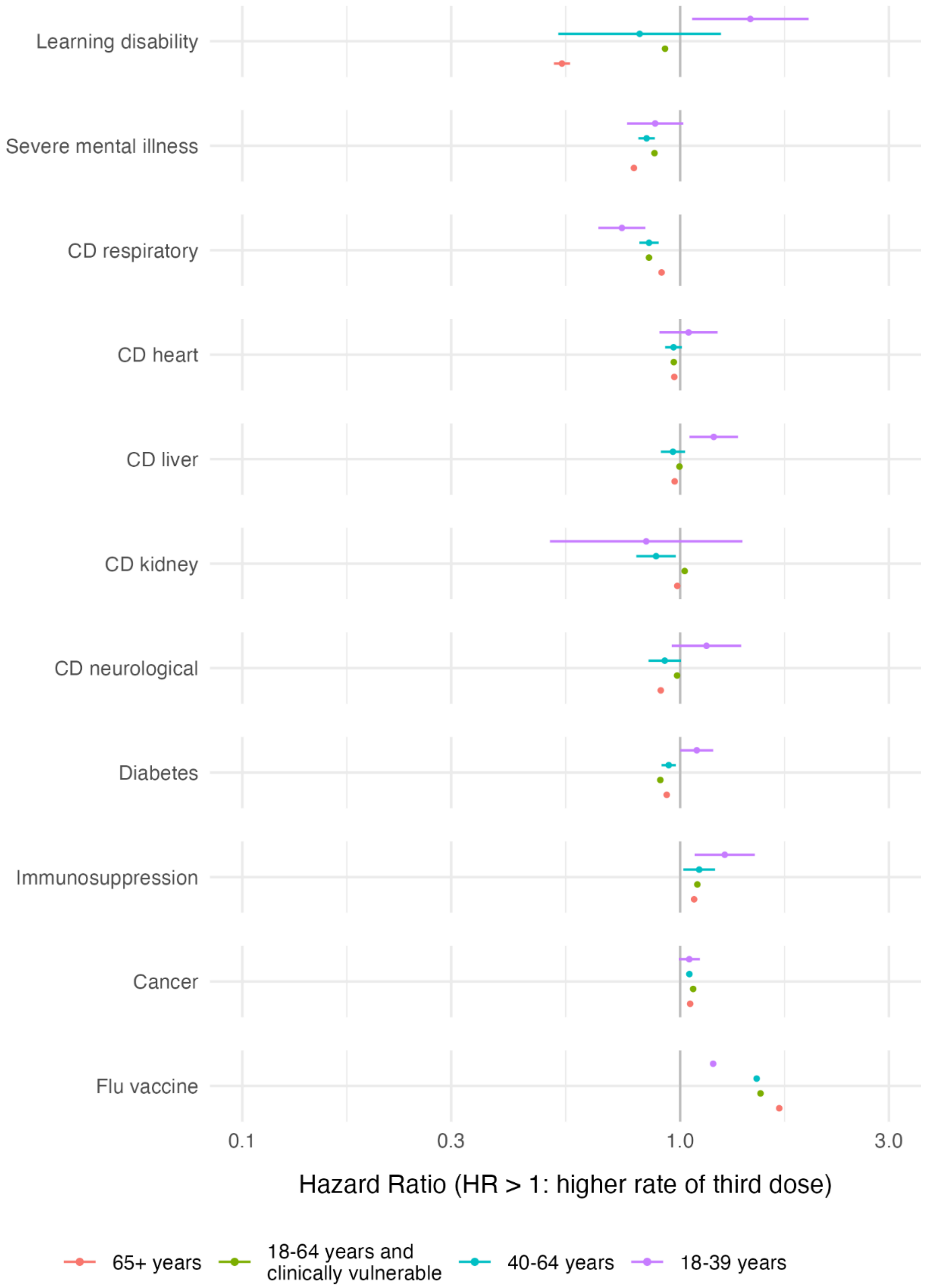

Web Figure 4. Hazard ratios for uptake of third dose. All variables defined as ever occurring before baseline, except for cancer (must have code in three years prior to baseline), and flu vaccine (received in 5 years before baseline). CD = chronic disease.

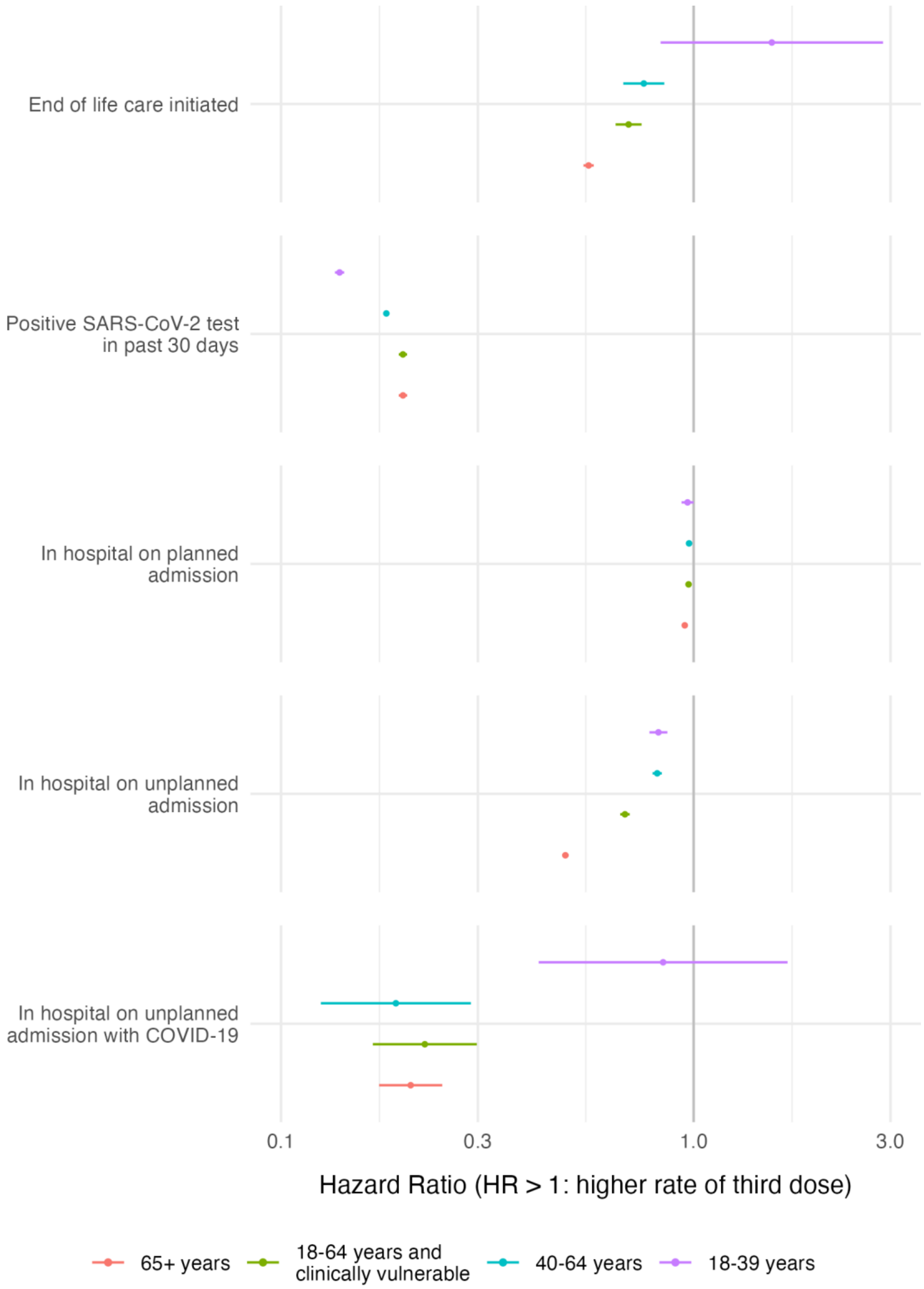

Web Figure 5. Hazard ratios for uptake of third dose. All variables are time-updating.

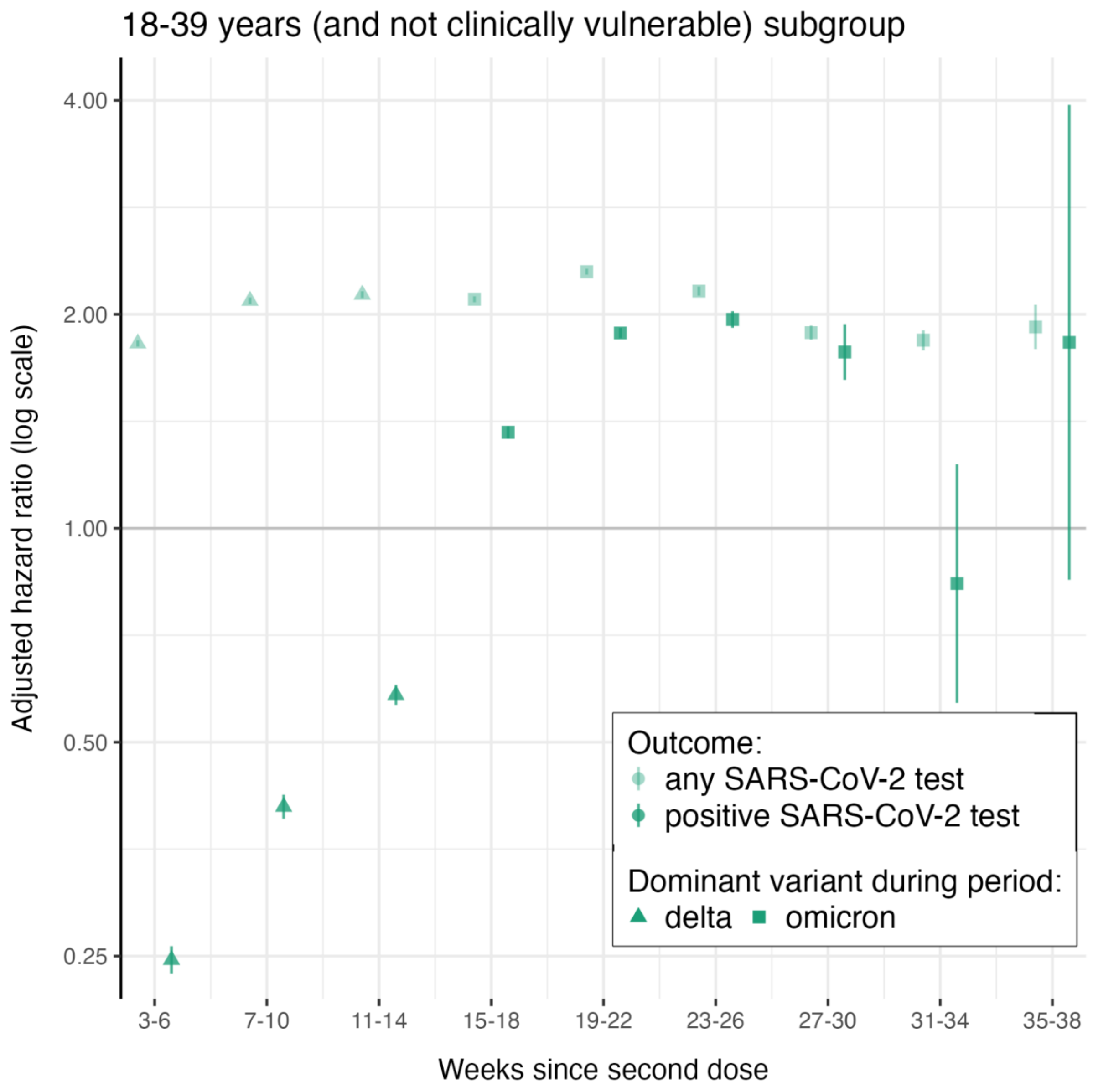

Web Figure 6. Hazard ratios for SARS-CoV-2 test and positive SARS-CoV-2 test in the 18-39 years subgroup. Follow-up was censored for both outcomes at the date of the first positive SARS-CoV-2 test. Only the first SARS-CoV-2 test in each comparison period (defined by weeks since second dose) was counted. The calendar time corresponding to each comparison period can be inferred from Web Figure 1.

Web Tables 1-21

Web Table 1. Variable definitions

Use	Name	Description
Stratification variable in all Cox models for estimating vaccine effectiveness	JCVI group	Vaccine priority group defined by the UK Joint Committee on Vaccination and Immunisation (JCVI)
	Eligibility date	Date of eligibility for primary vaccine course
	Region	NHS Region of England
Demographic covariate in adjusted Cox model	Age	Age on 31 March 2021
	Sex	Male, Female
	Index of Multiple Deprivation	English Index of Multiple Deprivation (IMD) quintile
	Ethnicity	Census categories: White, Black, South Asian, Mixed, Other
Clinical covariate in adjusted Cox model	BMI	Body Mass Index (BMI): Not Obese, Obese I (30-34.9 kg/m2), Obese II (35-39.9 kg/m2), Obese III (340 kg/m2),
	Learning disability	Yes, no
	Serious mental illness	Yes, no
	Morbidity count	Number of morbidities in the following categories: chronic respiratory disease, chronic heart disease, chronic liver disease, chronic kidney disease, chronic neurological disease, diabetes, immunosuppression.
	Flu vaccine in previous 5 years	Yes, no
	Number of previous SARS-CoV-2 tests	Number of tests taken between 2022-05-18 and "Eligibility date": 0, 1, 2, ≥3
	Pregnancy	Yes, no

Web Table 2. Counts and hazard ratios (HR) for Any SARS-CoV-2 test the 18-39 years subgroup

	Unvaccinated		BNT162b2			
weeks since second dose	n	events	n	events	unadjusted HR	adjusted HR
3-6	678,657	101,213	701,554	119,784	2.35 (2.32,2.37)	1.82 (1.80,1.84)
7-10	637,630	86,037	694,400	120,169	2.71 (2.69,2.74)	2.09 (2.07,2.11)
11-14	609,763	81,767	687,043	117,593	2.81 (2.78,2.84)	2.13 (2.11,2.15)
15-18	586,173	100,380	676,578	152,446	2.76 (2.73,2.78)	2.10 (2.08,2.12)
19-22	561,239	100,191	543,718	145,754	3.02 (2.99,3.04)	2.30 (2.27,2.32)
23-26	523,810	63,567	245,630	41,895	2.83 (2.79,2.87)	2.16 (2.12,2.19)
27-30	494,970	34,517	164,507	12,439	2.43 (2.38,2.48)	1.88 (1.84,1.93)
31-34	484,484	18,284	134,981	6,174	2.39 (2.32,2.47)	1.84 (1.78,1.90)
35-38	310,051	5,299	38,731	1,141	2.68 (2.51,2.87)	1.92 (1.79,2.06)

Web Table 3. Counts and hazard ratios (HR) for non-COVID-19 death the 18-39 years subgroup

	Unvaccinated		BNT162b2			
weeks since second dose	n	events	n	events	unadjusted HR	adjusted HR
3-6	685,895	28	706,468	7	0.66 (0.27,1.59)	0.64 (0.23,1.76)
7-10	654,024	28	702,009	7	0.18 (0.05,0.64)	0.16 (0.04,0.59)
11-14	631,344	21	698,761	7	0.57 (0.21,1.53)	0.67 (0.27,1.68)
15-18	614,145	21	695,639	14	0.65 (0.29,1.45)	0.64 (0.27,1.53)
19-22	596,470	21	584,493	7	0.36 (0.09,1.48)	0.34 (0.08,1.47)
23-26	576,205	21	305,452	7	0.41 (0.10,1.71)	0.34 (0.07,1.60)
27-30	559,132	14	219,408	7		
31-34	550,060	7	179,095	7		
35-38	351,512	7	52,325	0		

Web Table 4. Counts and hazard ratios (HR) for positive SARS-CoV-2 test the 18-39 years subgroup

	Unvaccinated		BNT162b2			
weeks since second dose	n	events	n	events	unadjusted HR	adjusted HR
3-6	678,657	16,450	701,554	2,744	0.31 (0.30,0.32)	0.25 (0.24,0.26)
7-10	637,630	14,203	694,400	4,151	0.52 (0.50,0.54)	0.41 (0.39,0.42)
11-14	609,763	16,688	687,043	7,420	0.75 (0.73,0.78)	0.58 (0.56,0.60)
15-18	586,173	29,519	676,578	27,195	1.71 (1.68,1.74)	1.36 (1.34,1.39)
19-22	561,239	35,399	543,718	42,980	2.32 (2.29,2.36)	1.88 (1.85,1.91)
23-26	523,810	18,200	245,630	12,880	2.52 (2.46,2.58)	1.97 (1.91,2.02)
27-30	494,970	3,325	164,507	707	2.38 (2.18,2.59)	1.77 (1.62,1.94)
31-34	484,484	196	134,981	42	1.11 (0.77,1.60)	0.84 (0.57,1.23)
35-38	310,051	56	38,731	14	2.54 (1.23,5.23)	1.83 (0.85,3.94)

Web Table 5. Counts and hazard ratios (HR) for COVID-19 death the 18-39 years subgroup

	Unvaccinated		BNT162b2			
weeks since second dose	n	events	n	events	unadjusted HR	adjusted HR
3-6	685,895	14	706,468	0		
7-10	654,024	14	702,009	0		
11-14	631,344	14	698,761	0		
15-18	614,145	14	695,639	0		
19-22	596,470	7	584,493	0		
23-26	576,205	7	305,452	7		
27-30	559,132	7	219,408	0		
31-34	550,060	7	179,095	0		
35-38	351,512	0	52,325	0		

Web Table 6. Counts and hazard ratios (HR) for COVID-19 hospitalisation the 18-39 years subgroup

	Unvaccinated		BNT162b2			
weeks since second dose	n	events	n	events	unadjusted HR	adjusted HR
3-6	685,622	763	706,454	21	0.04 (0.02,0.06)	0.04 (0.03,0.07)
7-10	653,359	560	701,974	28	0.09 (0.06,0.14)	0.10 (0.07,0.15)
11-14	630,420	574	698,698	42	0.11 (0.08,0.15)	0.10 (0.07,0.15)
15-18	613,081	665	695,541	70	0.18 (0.14,0.23)	0.17 (0.13,0.23)
19-22	595,175	791	584,339	126	0.28 (0.23,0.34)	0.30 (0.24,0.37)
23-26	574,707	630	305,249	84	0.50 (0.40,0.64)	0.49 (0.38,0.63)
27-30	557,340	392	219,170	56	0.63 (0.46,0.85)	0.56 (0.41,0.77)
31-34	548,149	224	178,864	42	0.89 (0.63,1.26)	0.69 (0.48,0.99)
35-38	350,091	56	52,241	14	1.97 (0.96,4.05)	1.48 (0.69,3.17)

Web Table 7. Counts and hazard ratios (HR) for Any SARS-CoV-2 test the 40-64 years subgroup

	Unvaccinated		ChAdOx1			
weeks since second dose	n	events	n	events	unadjusted HR	adjusted HR
3-6	314,615	34,706	1,500,366	246,358	3.81 (3.76,3.86)	2.83 (2.79,2.87)
7-10	296,149	34,566	1,489,642	254,555	3.53 (3.48,3.57)	2.67 (2.64,2.71)
11-14	282,863	32,788	1,474,256	255,353	3.75 (3.70,3.80)	2.86 (2.82,2.90)
15-18	273,161	30,786	1,454,971	279,734	4.15 (4.10,4.21)	3.15 (3.11,3.19)
19-22	265,405	31,465	1,432,242	289,226	4.28 (4.23,4.34)	3.26 (3.21,3.30)
23-26	258,083	33,460	1,377,586	296,072	4.42 (4.37,4.48)	3.40 (3.35,3.44)
27-30	249,795	30,898	934,052	211,099	3.73 (3.68,3.79)	2.89 (2.84,2.93)
31-34	238,924	21,350	160,083	19,117	2.68 (2.62,2.74)	2.21 (2.16,2.26)
35-38	231,945	13,125	114,191	7,343	2.31 (2.24,2.39)	1.90 (1.83,1.96)
39-42	228,557	7,266	89,033	4,340	2.49 (2.39,2.61)	2.05 (1.95,2.14)
43-46	92,120	2,541	48,832	1,407	2.84 (2.65,3.06)	2.28 (2.11,2.46)
47-50	3,255	28	63	7		

Web Table 8. Counts and hazard ratios (HR) for non-COVID-19 death the 40-64 years subgroup

	Unvaccinated		ChAdOx1			
weeks since second dose	n	events	n	events	unadjusted HR	adjusted HR
3-6	315,329	98	1,504,090	91	0.25 (0.18,0.35)	0.27 (0.18,0.41)
7-10	299,250	77	1,502,452	119	0.39 (0.28,0.54)	0.41 (0.28,0.60)
11-14	288,862	84	1,500,744	126	0.41 (0.30,0.56)	0.45 (0.32,0.63)
15-18	281,512	63	1,499,148	161	0.75 (0.53,1.06)	0.82 (0.56,1.20)
19-22	276,241	77	1,497,076	168	0.57 (0.41,0.79)	0.60 (0.42,0.87)
23-26	271,425	70	1,469,447	168	0.56 (0.40,0.78)	0.66 (0.45,0.95)
27-30	266,399	70	1,027,222	140	0.58 (0.40,0.83)	0.65 (0.44,0.97)
31-34	260,659	70	207,333	63	1.33 (0.91,1.95)	1.23 (0.84,1.80)
35-38	256,767	63	149,324	42	1.41 (0.94,2.12)	1.22 (0.80,1.87)
39-42	253,722	42	113,442	35	1.60 (0.99,2.60)	1.52 (0.93,2.48)
43-46	100,436	21	59,577	14	2.13 (0.93,4.91)	2.05 (0.88,4.74)
47-50	3,465	0	70	0		

Web Table 9. Counts and hazard ratios (HR) for positive SARS-CoV-2 test the 40-64 years subgroup

	Unvaccinated		ChAdOx1			
weeks since second dose	n	events	n	events	unadjusted HR	adjusted HR
3-6	314,615	5,866	1,500,366	9,100	0.91 (0.88,0.95)	0.78 (0.75,0.81)
7-10	296,149	5,985	1,489,642	13,692	1.06 (1.02,1.09)	0.92 (0.89,0.96)
11-14	282,863	5,670	1,474,256	17,731	1.48 (1.43,1.54)	1.27 (1.22,1.32)
15-18	273,161	5,887	1,454,971	20,734	1.83 (1.77,1.89)	1.52 (1.46,1.57)
19-22	265,405	6,860	1,432,242	29,239	2.17 (2.10,2.23)	1.77 (1.71,1.83)
23-26	258,083	9,884	1,377,586	43,428	2.60 (2.54,2.67)	2.18 (2.13,2.24)
27-30	249,795	9,632	934,052	33,656	2.37 (2.30,2.44)	2.07 (2.01,2.14)
31-34	238,924	4,417	160,083	5,320	2.35 (2.24,2.46)	2.03 (1.93,2.13)
35-38	231,945	861	114,191	322	1.95 (1.69,2.25)	1.60 (1.38,1.86)
39-42	228,557	70	89,033	42	1.71 (1.09,2.69)	1.29 (0.80,2.10)
43-46	92,120	28	48,832	7	0.80 (0.36,1.79)	0.59 (0.23,1.47)
47-50	3,255	0	63	0		

Web Table 10. Counts and hazard ratios (HR) for COVID-19 death the 40-64 years subgroup

	Unvaccinated		ChAdOx1			
weeks since second dose	n	events	n	events	unadjusted HR	adjusted HR
3-6	315,329	14	1,504,090	7		
7-10	299,250	21	1,502,452	7		
11-14	288,862	49	1,500,744	7	0.02 (0.01,0.05)	0.01 (0.00,0.04)
15-18	281,512	35	1,499,148	7	0.04 (0.02,0.09)	0.04 (0.01,0.09)
19-22	276,241	35	1,497,076	14	0.06 (0.03,0.13)	0.05 (0.02,0.10)
23-26	271,425	42	1,469,447	21	0.07 (0.04,0.14)	0.06 (0.03,0.14)
27-30	266,399	42	1,027,222	21	0.14 (0.07,0.28)	0.10 (0.05,0.24)
31-34	260,659	35	207,333	7	0.29 (0.12,0.66)	0.26 (0.11,0.64)
35-38	256,767	14	149,324	7		
39-42	253,722	7	113,442	7		
43-46	100,436	7	59,577	0		
47-50	3,465	0	70	0		

Web Table 11. Counts and hazard ratios (HR) for COVID-19 hospitalisation the 40-64 years subgroup

	Unvaccinated		ChAdOx1			
weeks since second dose	n	events	n	events	unadjusted HR	adjusted HR
3-6	315,294	511	1,504,055	63	0.06 (0.04,0.08)	0.05 (0.04,0.07)
7-10	299,026	588	1,502,354	112	0.06 (0.05,0.07)	0.05 (0.04,0.07)
11-14	288,365	602	1,500,534	126	0.05 (0.04,0.07)	0.04 (0.03,0.06)
15-18	280,805	525	1,498,819	175	0.10 (0.09,0.13)	0.09 (0.07,0.11)
19-22	275,310	504	1,496,586	210	0.14 (0.12,0.17)	0.12 (0.10,0.15)
23-26	270,340	553	1,468,761	322	0.18 (0.15,0.21)	0.14 (0.12,0.17)
27-30	265,125	497	1,026,424	273	0.19 (0.16,0.23)	0.18 (0.15,0.23)
31-34	259,210	273	206,864	119	0.74 (0.58,0.95)	0.62 (0.47,0.81)
35-38	255,199	154	148,925	63	1.02 (0.76,1.39)	0.83 (0.61,1.15)
39-42	252,147	70	113,092	42	1.48 (0.96,2.28)	1.12 (0.70,1.80)
43-46	99,624	28	59,332	7	0.76 (0.34,1.69)	0.56 (0.22,1.41)
47-50	3,423	0	70	0		

Web Table 12. Counts and hazard ratios (HR) for Any SARS-CoV-2 test the 18-64 years and clinically vulnerable subgroup

	Unvaccinated		BNT162b2				ChAdOx1			
weeks since second dose	n	events	n	events	unadjusted HR	adjusted HR	n	events	unadjusted HR	adjusted HR
3-6	152,572	20,384	373,863	48,503	2.23 (2.19,2.27)	2.04 (2.00,2.09)	658,742	92,666	2.49 (2.45,2.53)	2.23 (2.19,2.27)
7-10	135,653	20,580	373,254	60,256	2.11 (2.07,2.14)	1.95 (1.91,1.99)	656,404	109,935	2.36 (2.32,2.40)	2.11 (2.07,2.15)
11-14	122,136	19,873	371,308	57,442	2.00 (1.96,2.03)	1.83 (1.79,1.87)	649,866	103,271	2.20 (2.17,2.24)	1.98 (1.95,2.02)
15-18	113,092	17,983	368,396	58,737	1.97 (1.93,2.01)	1.78 (1.74,1.82)	641,039	109,018	2.26 (2.22,2.30)	2.01 (1.97,2.04)
19-22	107,212	16,842	364,595	64,687	2.26 (2.22,2.30)	2.00 (1.96,2.04)	631,421	116,522	2.54 (2.50,2.59)	2.20 (2.16,2.24)
23-26	102,802	15,365	359,415	65,919	2.47 (2.42,2.52)	2.12 (2.07,2.17)	618,121	115,262	2.75 (2.71,2.80)	2.35 (2.30,2.39)
27-30	98,840	16,751	332,892	61,334	2.41 (2.37,2.46)	2.11 (2.07,2.16)	552,272	109,956	2.58 (2.53,2.62)	2.24 (2.20,2.28)
31-34	94,864	17,528	98,028	18,431	1.88 (1.84,1.92)	1.76 (1.71,1.80)	161,931	30,779	2.02 (1.98,2.06)	1.90 (1.85,1.94)
35-38	89,509	13,839	34,265	5,341	1.81 (1.75,1.88)	1.65 (1.59,1.71)	72,324	10,073	1.92 (1.86,1.97)	1.74 (1.69,1.79)
39-42	83,545	8,449	24,773	2,142	1.73 (1.65,1.82)	1.53 (1.46,1.62)	56,742	4,697	1.95 (1.88,2.03)	1.75 (1.67,1.82)
43-46	81,578	5,236	23,184	1,603	1.69 (1.59,1.79)	1.49 (1.40,1.58)	53,480	3,185	1.89 (1.80,1.99)	1.69 (1.60,1.78)
47-50	80,619	2,135	14,091	434	1.91 (1.71,2.13)	1.74 (1.55,1.95)	14,070	693	2.04 (1.84,2.26)	1.81 (1.63,2.02)

Web Table 13. Counts and hazard ratios (HR) for non-COVID-19 death the 18-64 years and clinically vulnerable subgroup

	Unvaccinated		BNT162b2				ChAdOx1			
weeks since second dose	n	events	n	events	unadjusted HR	adjusted HR	n	events	unadjusted HR	adjusted HR
3-6	152,677	147	373,961	133	0.69 (0.53,0.91)	0.45 (0.33,0.61)	659,050	196	0.51 (0.40,0.64)	0.34 (0.26,0.44)
7-10	136,024	154	373,492	133	0.56 (0.42,0.75)	0.35 (0.25,0.49)	658,112	245	0.52 (0.42,0.64)	0.33 (0.25,0.43)
11-14	123,508	133	372,974	147	0.60 (0.46,0.79)	0.33 (0.24,0.45)	657,132	287	0.60 (0.48,0.75)	0.36 (0.28,0.47)
15-18	116,291	126	372,449	154	0.71 (0.55,0.92)	0.41 (0.30,0.56)	656,054	308	0.78 (0.62,0.98)	0.57 (0.44,0.75)
19-22	112,000	119	371,770	147	0.65 (0.49,0.86)	0.42 (0.30,0.57)	654,787	301	0.73 (0.57,0.93)	0.49 (0.38,0.64)
23-26	108,374	112	369,894	182	0.84 (0.63,1.11)	0.47 (0.35,0.65)	651,259	315	0.86 (0.66,1.11)	0.58 (0.44,0.77)
27-30	105,672	105	347,928	175	0.89 (0.68,1.17)	0.62 (0.44,0.87)	595,651	322	0.90 (0.71,1.14)	0.64 (0.49,0.85)
31-34	102,725	105	107,639	112	1.84 (1.35,2.52)	1.20 (0.87,1.65)	187,355	175	1.56 (1.18,2.07)	1.18 (0.88,1.59)
35-38	99,547	77	40,432	63	2.95 (2.06,4.24)	2.24 (1.55,3.24)	90,699	98	2.13 (1.56,2.93)	1.63 (1.19,2.23)
39-42	96,278	77	30,891	42	2.40 (1.57,3.67)	1.75 (1.14,2.67)	73,885	63	2.06 (1.41,2.99)	1.82 (1.23,2.68)
43-46	94,864	63	28,903	28	1.73 (1.05,2.83)	1.28 (0.76,2.14)	69,664	49	1.79 (1.23,2.62)	1.42 (0.95,2.13)
47-50	93,590	21	17,507	7	2.41 (0.98,5.93)	2.02 (0.85,4.79)	18,487	14	1.86 (0.76,4.59)	1.51 (0.61,3.77)

Web Table 14. Counts and hazard ratios (HR) for positive SARS-CoV-2 test the 18-64 years and clinically vulnerable subgroup

	Unvaccinated		BNT162b2				ChAdOx1			
weeks since second dose	n	events	n	events	unadjusted HR	adjusted HR	n	events	unadjusted HR	adjusted HR
3-6	152,572	1,624	373,863	147	0.17 (0.14,0.21)	0.22 (0.18,0.27)	658,742	1,407	0.60 (0.55,0.65)	0.74 (0.67,0.80)
7-10	135,653	3,374	373,254	1,435	0.31 (0.29,0.34)	0.39 (0.36,0.42)	656,404	5,572	0.64 (0.61,0.67)	0.77 (0.73,0.81)
11-14	122,136	3,983	371,308	2,394	0.39 (0.36,0.41)	0.45 (0.42,0.48)	649,866	7,784	0.73 (0.70,0.76)	0.85 (0.81,0.89)
15-18	113,092	2,989	368,396	3,150	0.56 (0.53,0.59)	0.59 (0.55,0.63)	641,039	8,414	0.98 (0.93,1.02)	1.03 (0.98,1.09)
19-22	107,212	2,695	364,595	3,374	0.80 (0.75,0.85)	0.80 (0.75,0.86)	631,421	9,926	1.24 (1.19,1.30)	1.21 (1.15,1.27)
23-26	102,802	2,940	359,415	5,180	0.94 (0.89,0.99)	0.92 (0.86,0.97)	618,121	12,901	1.43 (1.37,1.50)	1.38 (1.31,1.44)
27-30	98,840	4,095	332,892	4,928	0.84 (0.80,0.89)	0.86 (0.81,0.91)	552,272	12,957	1.29 (1.24,1.35)	1.32 (1.26,1.38)
31-34	94,864	6,048	98,028	4,130	1.33 (1.27,1.39)	1.37 (1.31,1.44)	161,931	9,163	1.64 (1.58,1.70)	1.67 (1.61,1.73)
35-38	89,509	4,130	34,265	1,848	1.69 (1.60,1.79)	1.59 (1.50,1.70)	72,324	2,898	1.69 (1.60,1.78)	1.60 (1.51,1.70)
39-42	83,545	707	24,773	140	1.55 (1.27,1.90)	1.39 (1.13,1.71)	56,742	322	1.65 (1.42,1.92)	1.53 (1.31,1.79)
43-46	81,578	203	23,184	35	0.84 (0.57,1.25)	0.64 (0.43,0.97)	53,480	77	0.96 (0.73,1.27)	0.76 (0.57,1.01)
47-50	80,619	84	14,091	14	0.92 (0.49,1.75)	0.72 (0.37,1.41)	14,070	28	1.07 (0.67,1.71)	0.86 (0.52,1.42)

Web Table 15. Counts and hazard ratios (HR) for COVID-19 death the 18-64 years and clinically vulnerable subgroup

	Unvaccinated		BNT162b2				ChAdOx1			
weeks since second dose	n	events	n	events	unadjusted HR	adjusted HR	n	events	unadjusted HR	adjusted HR
3-6	152,677	14	373,961	0			659,050	7		
7-10	136,024	28	373,492	0			658,112	7	0.07 (0.02,0.23)	0.04 (0.01,0.13)
11-14	123,508	63	372,974	7			657,132	21	0.12 (0.07,0.20)	0.09 (0.05,0.16)
15-18	116,291	63	372,449	14	0.07 (0.03,0.14)	0.04 (0.02,0.08)	656,054	28	0.14 (0.09,0.23)	0.08 (0.05,0.13)
19-22	112,000	70	371,770	14	0.12 (0.07,0.23)	0.07 (0.04,0.14)	654,787	35	0.15 (0.10,0.24)	0.09 (0.06,0.15)
23-26	108,374	49	369,894	14	0.14 (0.07,0.28)	0.07 (0.03,0.14)	651,259	42	0.22 (0.14,0.34)	0.11 (0.07,0.17)
27-30	105,672	63	347,928	21	0.16 (0.09,0.28)	0.08 (0.04,0.15)	595,651	56	0.24 (0.16,0.36)	0.12 (0.08,0.19)
31-34	102,725	56	107,639	14	0.33 (0.16,0.65)	0.19 (0.10,0.37)	187,355	28	0.43 (0.26,0.69)	0.29 (0.17,0.50)
35-38	99,547	42	40,432	14	0.81 (0.38,1.73)	0.63 (0.30,1.36)	90,699	14	0.78 (0.41,1.48)	0.57 (0.30,1.10)
39-42	96,278	21	30,891	7			73,885	7	0.40 (0.14,1.18)	0.37 (0.13,1.07)
43-46	94,864	14	28,903	7			69,664	7	0.66 (0.18,2.45)	0.50 (0.11,2.25)
47-50	93,590	7	17,507	7			18,487	7		

Web Table 16. Counts and hazard ratios (HR) for COVID-19 hospitalisation the 18-64 years and clinically vulnerable subgroup

	Unvaccinated		BNT162b2				ChAdOx1			
weeks since second dose	n	events	n	events	unadjusted HR	adjusted HR	n	events	unadjusted HR	adjusted HR
3-6	152,670	189	373,947	7	0.04 (0.02,0.10)	0.03 (0.01,0.08)	659,036	28	0.09 (0.06,0.14)	0.07 (0.04,0.11)
7-10	135,975	448	373,471	28	0.06 (0.04,0.08)	0.04 (0.03,0.06)	658,077	161	0.14 (0.12,0.17)	0.11 (0.09,0.14)
11-14	123,340	630	372,932	63	0.06 (0.05,0.08)	0.05 (0.04,0.06)	656,943	308	0.17 (0.14,0.19)	0.13 (0.11,0.15)
15-18	115,857	490	372,344	105	0.12 (0.10,0.15)	0.09 (0.07,0.11)	655,571	406	0.26 (0.23,0.30)	0.20 (0.17,0.24)
19-22	111,314	448	371,574	105	0.14 (0.11,0.17)	0.09 (0.07,0.11)	653,933	392	0.28 (0.24,0.33)	0.20 (0.17,0.23)
23-26	107,583	441	369,607	133	0.17 (0.14,0.21)	0.10 (0.08,0.13)	650,055	518	0.35 (0.30,0.40)	0.25 (0.21,0.29)
27-30	104,713	455	347,543	154	0.19 (0.16,0.23)	0.12 (0.10,0.15)	594,104	497	0.33 (0.29,0.38)	0.24 (0.21,0.29)
31-34	101,661	511	107,373	119	0.38 (0.31,0.47)	0.28 (0.22,0.35)	186,298	294	0.54 (0.46,0.63)	0.42 (0.36,0.50)
35-38	98,322	406	40,229	105	1.01 (0.80,1.26)	0.80 (0.64,1.01)	89,964	196	0.95 (0.79,1.15)	0.83 (0.69,1.01)
39-42	94,913	245	30,674	49	1.14 (0.80,1.62)	0.91 (0.64,1.30)	73,213	112	1.21 (0.95,1.54)	1.02 (0.80,1.31)
43-46	93,401	203	28,672	35	0.78 (0.53,1.15)	0.61 (0.41,0.90)	68,957	84	0.87 (0.66,1.14)	0.70 (0.53,0.92)
47-50	92,078	84	17,353	14	0.92 (0.50,1.71)	0.72 (0.38,1.38)	18,193	28	0.99 (0.62,1.60)	0.79 (0.48,1.30)

Web Table 17. Counts and hazard ratios (HR) for Any SARS-CoV-2 test the 65+ years subgroup

	Unvaccinated		BNT162b2				ChAdOx1			
weeks since second dose	n	events	n	events	unadjusted HR	adjusted HR	n	events	unadjusted HR	adjusted HR
3-6	73,227	5,810	845,880	66,556	2.21 (2.15,2.28)	1.61 (1.56,1.67)	1,117,746	102,060	2.97 (2.88,3.06)	2.09 (2.02,2.16)
7-10	66,773	5,439	844,375	74,410	2.55 (2.48,2.63)	1.87 (1.80,1.93)	1,115,828	113,463	3.12 (3.02,3.22)	2.19 (2.12,2.27)
11-14	64,008	5,516	842,492	81,816	2.55 (2.48,2.63)	1.87 (1.80,1.93)	1,112,818	122,906	3.07 (2.98,3.17)	2.19 (2.12,2.27)
15-18	61,215	5,558	839,524	81,564	2.48 (2.40,2.56)	1.84 (1.78,1.91)	1,107,372	120,995	3.00 (2.91,3.10)	2.16 (2.09,2.23)
19-22	59,906	5,537	835,345	87,339	2.61 (2.53,2.69)	1.94 (1.87,2.00)	1,099,861	134,113	3.18 (3.09,3.28)	2.29 (2.22,2.37)
23-26	57,946	5,376	829,892	96,061	2.87 (2.79,2.96)	2.14 (2.07,2.21)	1,090,474	146,216	3.45 (3.35,3.56)	2.45 (2.38,2.53)
27-30	57,239	5,551	788,963	94,430	2.87 (2.78,2.95)	2.15 (2.08,2.22)	1,017,905	140,140	3.47 (3.36,3.57)	2.50 (2.42,2.58)
31-34	55,391	5,726	163,443	18,942	2.41 (2.32,2.49)	1.89 (1.82,1.97)	174,342	21,119	2.70 (2.61,2.80)	2.10 (2.02,2.18)
35-38	54,075	5,159	38,528	5,327	2.85 (2.73,2.98)	2.24 (2.13,2.35)	45,276	5,852	3.05 (2.92,3.19)	2.41 (2.30,2.53)
39-42	51,772	4,109	18,256	2,401	3.31 (3.13,3.51)	2.66 (2.50,2.83)	25,914	2,576	3.26 (3.07,3.46)	2.65 (2.49,2.82)
43-46	50,785	3,276	13,762	1,393	3.27 (3.05,3.51)	2.65 (2.46,2.85)	22,456	1,701	3.04 (2.83,3.26)	2.53 (2.35,2.72)
47-50	49,357	2,163	11,767	931	3.30 (3.03,3.60)	2.67 (2.43,2.92)	17,122	1,015	3.15 (2.87,3.46)	2.59 (2.36,2.85)

Web Table 18. Counts and hazard ratios (HR) for non-COVID-19 death the 65+ years subgroup

	Unvaccinated		BNT162b2				ChAdOx1			
weeks since second dose	n	events	n	events	unadjusted HR	adjusted HR	n	events	unadjusted HR	adjusted HR
3-6	73,269	385	845,992	854	0.31 (0.27,0.36)	0.28 (0.24,0.34)	1,117,914	812	0.43 (0.36,0.51)	0.34 (0.28,0.41)
7-10	66,829	301	844,585	1,092	0.44 (0.38,0.51)	0.40 (0.33,0.47)	1,116,241	973	0.60 (0.49,0.72)	0.50 (0.41,0.62)
11-14	64,106	280	843,017	1,274	0.54 (0.47,0.62)	0.51 (0.43,0.61)	1,114,435	1,148	0.62 (0.52,0.74)	0.60 (0.50,0.73)
15-18	61,474	259	841,127	1,337	0.57 (0.49,0.66)	0.51 (0.42,0.62)	1,112,356	1,253	0.77 (0.64,0.92)	0.65 (0.53,0.79)
19-22	60,319	259	839,195	1,393	0.61 (0.52,0.71)	0.60 (0.50,0.72)	1,110,249	1,190	0.65 (0.54,0.78)	0.65 (0.53,0.79)
23-26	58,653	273	836,731	1,568	0.58 (0.50,0.66)	0.52 (0.44,0.62)	1,107,232	1,372	0.72 (0.61,0.84)	0.68 (0.56,0.82)
27-30	58,072	280	799,386	1,582	0.65 (0.57,0.75)	0.61 (0.51,0.73)	1,042,377	1,400	0.76 (0.64,0.89)	0.70 (0.58,0.84)
31-34	56,525	266	169,659	1,057	1.64 (1.40,1.91)	1.25 (1.05,1.49)	187,117	791	1.94 (1.65,2.28)	1.41 (1.19,1.69)
35-38	55,496	287	40,782	567	3.33 (2.82,3.92)	2.40 (2.02,2.85)	49,266	455	3.40 (2.87,4.03)	2.41 (2.03,2.87)
39-42	53,676	287	19,481	266	3.40 (2.83,4.08)	2.72 (2.24,3.30)	28,378	203	3.14 (2.54,3.87)	2.53 (2.05,3.13)
43-46	53,060	252	14,868	126	2.63 (2.07,3.35)	2.11 (1.63,2.73)	24,717	154	3.31 (2.59,4.22)	2.67 (2.08,3.42)
47-50	51,772	175	12,782	91	2.53 (1.90,3.37)	2.00 (1.48,2.70)	18,816	70	2.36 (1.68,3.31)	1.87 (1.33,2.64)

Web Table 19. Counts and hazard ratios (HR) for positive SARS-CoV-2 test the 65+ years subgroup

	Unvaccinated		BNT162b2				ChAdOx1			
weeks since second dose	n	events	n	events	unadjusted HR	adjusted HR	n	events	unadjusted HR	adjusted HR
3-6	73,227	105	845,880	105	0.23 (0.16,0.31)	0.23 (0.16,0.33)	1,117,746	252	0.59 (0.45,0.79)	0.53 (0.39,0.71)
7-10	66,773	252	844,375	322	0.37 (0.30,0.45)	0.30 (0.24,0.38)	1,115,828	1,211	0.69 (0.59,0.81)	0.62 (0.52,0.74)
11-14	64,008	392	842,492	1,099	0.52 (0.46,0.60)	0.43 (0.37,0.50)	1,112,818	3,423	0.89 (0.79,1.00)	0.75 (0.66,0.86)
15-18	61,215	595	839,524	2,303	0.62 (0.56,0.69)	0.53 (0.47,0.60)	1,107,372	5,509	0.92 (0.84,1.02)	0.79 (0.71,0.87)
19-22	59,906	581	835,345	3,115	0.77 (0.69,0.85)	0.67 (0.59,0.75)	1,099,861	6,545	1.19 (1.08,1.31)	1.00 (0.90,1.12)
23-26	57,946	602	829,892	4,158	1.08 (0.98,1.19)	0.87 (0.78,0.97)	1,090,474	8,967	1.60 (1.46,1.76)	1.24 (1.12,1.36)
27-30	57,239	770	788,963	4,900	0.94 (0.86,1.02)	0.79 (0.71,0.87)	1,017,905	9,380	1.26 (1.16,1.37)	1.08 (0.98,1.18)
31-34	55,391	1,015	163,443	1,085	0.90 (0.81,1.00)	0.80 (0.71,0.90)	174,342	1,834	1.38 (1.26,1.52)	1.19 (1.07,1.31)
35-38	54,075	1,127	38,528	518	1.23 (1.10,1.39)	1.03 (0.91,1.17)	45,276	952	1.64 (1.48,1.80)	1.37 (1.24,1.53)
39-42	51,772	777	18,256	322	1.78 (1.54,2.05)	1.47 (1.26,1.72)	25,914	308	1.91 (1.64,2.23)	1.54 (1.31,1.81)
43-46	50,785	469	13,762	154	1.75 (1.43,2.14)	1.34 (1.08,1.65)	22,456	98	1.60 (1.24,2.07)	1.23 (0.95,1.59)
47-50	49,357	273	11,767	63	1.31 (0.98,1.75)	0.96 (0.71,1.30)	17,122	70	1.44 (1.06,1.96)	1.12 (0.82,1.54)

Web Table 20. Counts and hazard ratios (HR) for COVID-19 death the 65+ years subgroup

	Unvaccinated		BNT162b2				ChAdOx1			
weeks since second dose	n	events	n	events	unadjusted HR	adjusted HR	n	events	unadjusted HR	adjusted HR
3-6	73,269	21	845,992	7	0.03 (0.01,0.08)	0.03 (0.01,0.08)	1,117,914	7		
7-10	66,829	21	844,585	7			1,116,241	7	0.05 (0.02,0.11)	0.07 (0.03,0.16)
11-14	64,106	42	843,017	14	0.05 (0.02,0.10)	0.05 (0.02,0.10)	1,114,435	35	0.10 (0.06,0.16)	0.09 (0.05,0.15)
15-18	61,474	77	841,127	42	0.08 (0.05,0.12)	0.06 (0.04,0.10)	1,112,356	84	0.10 (0.07,0.15)	0.08 (0.05,0.13)
19-22	60,319	105	839,195	98	0.11 (0.08,0.16)	0.10 (0.07,0.15)	1,110,249	133	0.12 (0.09,0.17)	0.11 (0.08,0.16)
23-26	58,653	98	836,731	112	0.13 (0.10,0.18)	0.13 (0.09,0.19)	1,107,232	133	0.17 (0.12,0.23)	0.15 (0.10,0.21)
27-30	58,072	91	799,386	133	0.19 (0.14,0.26)	0.16 (0.11,0.23)	1,042,377	175	0.25 (0.18,0.35)	0.21 (0.15,0.31)
31-34	56,525	105	169,659	77	0.39 (0.28,0.55)	0.28 (0.19,0.41)	187,117	119	0.66 (0.49,0.89)	0.44 (0.32,0.61)
35-38	55,496	126	40,782	56	0.79 (0.57,1.09)	0.56 (0.40,0.79)	49,266	56	0.84 (0.58,1.21)	0.59 (0.41,0.86)
39-42	53,676	119	19,481	35	0.96 (0.64,1.45)	0.70 (0.46,1.08)	28,378	35	1.38 (0.89,2.13)	0.97 (0.63,1.50)
43-46	53,060	98	14,868	21	0.98 (0.58,1.65)	0.68 (0.38,1.19)	24,717	14	1.23 (0.62,2.46)	0.96 (0.49,1.89)
47-50	51,772	56	12,782	14	0.90 (0.43,1.88)	0.67 (0.30,1.49)	18,816	7	0.94 (0.40,2.25)	0.77 (0.32,1.82)

Web Table 21. Counts and hazard ratios (HR) for COVID-19 hospitalisation the 65+ years subgroup

	Unvaccinated		BNT162b2				ChAdOx1			
weeks since second dose	n	events	n	events	unadjusted HR	adjusted HR	n	events	unadjusted HR	adjusted HR
3-6	73,248	70	845,964	28	0.08 (0.05,0.13)	0.08 (0.05,0.14)	1,117,900	42	0.12 (0.08,0.20)	0.14 (0.08,0.25)
7-10	66,794	112	844,536	35	0.07 (0.04,0.10)	0.06 (0.04,0.10)	1,116,185	91	0.09 (0.07,0.13)	0.09 (0.06,0.13)
11-14	64,050	196	842,940	126	0.10 (0.08,0.14)	0.10 (0.08,0.14)	1,114,295	343	0.16 (0.13,0.19)	0.15 (0.12,0.19)
15-18	61,355	322	840,945	308	0.13 (0.11,0.15)	0.12 (0.09,0.15)	1,111,915	602	0.17 (0.14,0.20)	0.16 (0.13,0.19)
19-22	60,123	329	838,754	413	0.16 (0.13,0.18)	0.15 (0.12,0.18)	1,109,290	763	0.23 (0.20,0.27)	0.22 (0.18,0.26)
23-26	58,310	315	835,975	581	0.23 (0.19,0.26)	0.21 (0.18,0.25)	1,105,643	861	0.30 (0.25,0.35)	0.26 (0.22,0.31)
27-30	57,673	364	798,217	686	0.23 (0.20,0.26)	0.20 (0.17,0.24)	1,040,172	1,008	0.29 (0.25,0.33)	0.26 (0.22,0.31)
31-34	56,007	427	168,616	301	0.40 (0.34,0.48)	0.36 (0.29,0.44)	185,465	399	0.54 (0.46,0.63)	0.45 (0.38,0.54)
35-38	54,859	511	40,061	189	0.75 (0.62,0.90)	0.60 (0.50,0.74)	48,293	252	0.93 (0.79,1.10)	0.70 (0.59,0.83)
39-42	52,885	441	19,012	126	1.06 (0.86,1.31)	0.87 (0.70,1.09)	27,671	126	1.14 (0.92,1.43)	0.92 (0.73,1.15)
43-46	52,101	343	14,490	84	1.31 (1.01,1.68)	0.97 (0.75,1.27)	24,087	91	1.43 (1.10,1.86)	1.07 (0.82,1.41)
47-50	50,736	259	12,425	63	1.35 (1.01,1.81)	0.98 (0.73,1.34)	18,277	70	1.45 (1.07,1.97)	1.12 (0.82,1.53)

References

1. Horne EMF, Hulme WJ, Keogh RH, et al. Waning effectiveness of BNT162b2 and ChAdOx1 covid-19 vaccines over six months since second dose: OpenSAFELY cohort study using linked electronic health records. *BMJ*. 2022;378:e071249.

2. UK Health Security Agency. Omicron daily overview: 17 December 2021. Gov.uk. Published December 17, 2021. Accessed May 13, 2022. https://assets.publishing.service.gov.uk/government/uploads/system/uploads/attachment_data/file/1042100/20211217_OS_Daily_Omicron_Overview.pdf

3. NHS Digital. Data Security and Protection Toolkit. NHS Digital. Accessed April 30, 2020. https://digital.nhs.uk/data-and-information/looking-after-information/data-security-and-information-governance/data-security-and-protection-toolkit

4. NHS Digital. ISB1523: Anonymisation Standard for Publishing Health and Social Care Data. NHS Digital. Accessed April 30, 2020. https://digital.nhs.uk/data-and-information/information-standards/information-standards-and-data-collections-including-extractions/publications-and-notifications/standards-and-collections/isb1523-anonymisation-standard-for-publishing-health-and-social-care-data

5. Secretary of State for Health and Social Care. Coronavirus (COVID-19): notification to organisations to share information. UK Government. https://web.archive.org/web/20200421171727/https://www.gov.uk/government/publications/coronavirus-covid-19-notification-of-data-controllers-to-share-information

6. Secretary of State for Health and Social Care-UK Government. Coronavirus (COVID-19): notification to organisations to share information. Gov.uk. Published October 18, 2022. Accessed November 29, 2022. https://www.gov.uk/government/publications/coronavirus-covid-19-notification-to-organisations-to-share-information/coronavirus-covid-19-notice-under-regulation-34-of-the-health-service-control-of-patient-information-regulations-2002

7. Health Research Authority. Confidentiality Advisory Group. NHS Health Research Authority. Accessed November 29, 2022. https://www.hra.nhs.uk/about-us/committees-and-services/confidentiality-advisory-group/
